# The Transactions of the Provincial Medical and Surgical Association

**Published:** 1841-07

**Authors:** 


Art. XII.—*The Transactions of the Provincial Medical and Surgical Association.—London,* 1841. Vol. IX. 8vo, pp. 583.

The present annual volume of this flourishing Society consists, as usual, of four Parts, besides the proceedings of the association at the place of meeting for the year, Southampton, viz.: 1, Retrospective Addresses in Surgery and Medicine; 2, Papers on Medical Topography; 3, Original Essays and Cases; 4, Infirmary Reports and Statistics. In the First Part we have addresses by Mr. Dodd of Chichester, and Dr. Scott of Liverpool. In the Second Part, a memoir on the Medical topography of Shrewsbury and its neighbourhood, by Dr. Ogier Ward of Shrewsbury, and another (which belongs of right to Part V.) on the Statistics of rheumatism, by Dr. Lyon of Manchester. In the Third Part are—Observations on injuries of the skull and brain, by Mr. Banner of Liverpool; On moveable cartilaginous bodies in synovial and serous cavities, by Dr. Macartney of Dublin; A case of amputation of the lower jaw, by Mr. Dodd of Chichester; Cases of abscesses within the pelvis after labour, by Mr. Wainewright of Liverpool; Cases illustrating the pathology of biliary concretions, by Mr. Mallett of Bolton-le-Moors; and remarks on the influence of the depressing passions, by Dr. Walker of Huddersfield. In the Fourth Part we have—A report of the out-patients at the Birmingham Town Infirmary, by Mr. Ryland; A report of the cases at the Birmingham Eye Infirmary, by Mr. Middlemore; and Suggestions for a form of register for hospitals, by Dr. Cowan of Reading. We hope to return to this volume next quarter. Our limited space will only permit us at present to make one or two brief remarks on its contents which are fully as interesting as usual. The addresses of Mr. Dodd and of Dr. Scott are admirable productions and of considerable extent, the former occupying eighty-four and the latter ninety-six pages. Together they constitute a most valuable digest of the progress of medicine and surgery during the preceding year, and claim the especial notice of all members of the profession, being the only records of the kind published in this country. The perusal of Mr. Dodd's paper will leave no doubt in the mind of any one of the impolicy of the resolution passed at the Southampton meeting, of not having an annual address in surgery as well as in medicine. The argument contained in the two following propositions—1, That this address is full of matter of great importance to the practical provincial surgeon not to be obtained by him elsewhere; 2, That no *physician* in England could compose such an address—is, we think, conclusive in favour of there being an annual address in surgery; and we trust the mistake committed by the association last year at Southampton will be remedied this year at York. We are, indeed, of opinion that it would be highly expedient to subdivide the subject of these annual addresses still further, so as, in place of only one or two, to have even *three* annually, viz. one on Medicine, one on Surgery, and one on Anatomy and Physiology. By this means we should have every department of medical science more thoroughly explored, and its annual progress more clearly presented, while the individual addresses being shorter and delivered at different times during the session of the Association, would be listened to with more satisfaction and profit than can be expected under the present arrangements. The high character of the retrospective reviews and of the topographical and statistical memoirs contained in the present volume, especially Dr. Ward's *Shrewsbury*, adds force to our previous conviction that it is to the publication of papers of this sort that the transactions of the association should be principally devoted. There exist many other channels for communicating to the profession histories of cases and short essays of a scientific or practical nature; but it is only by an association of this kind, which has plenty of funds at its disposal, that long annual retrospects or topographical and statistical memoirs, comprising expensive tables, maps, and illustrative plates, can be given to the public. We will even go so far as to say that the association should confine itself to the printing of papers of this kind and reports on special subjects undertaken at its instance, essays that have obtained prizes from it, &c. &c. Into this category will come, of course, such memoirs as the admirable one of Mr. Ceeley, on Vaccination, in the last volume, the publication of which at the expense of the association is infinitely creditable to the council. We wish we could have added that the same liberality had been extended to Dr. Davidson and his excellent prize Essay, and that he had not been forced, by the rejection of the proposition to print it in the volume of Transactions, its natural *habitat*, to seek a place for it in the pages of a private journal.

